# 4-Allyl-6-bromo-2-phenyl-4*H*-imidazo[4,5-*b*]pyridine monohydrate

**DOI:** 10.1107/S1600536810025122

**Published:** 2010-07-03

**Authors:** Younès Ouzidan, Youssef Kandri Rodi, Hafid Zouihri, El Mokhtar Essassi, Seik Weng Ng

**Affiliations:** a Laboratoire de Chimie Organique Appliquée, Faculté des Sciences et Techniques, Université Sidi Mohamed Ben Abdallah, Fès, Morocco; b CNRST Division UATRS, Angle Allal Fassi/FAR, BP 8027 Hay Riad, Rabat, Morocco; c Laboratoire de Chimie Organique Hétérocyclique, Pôle de Compétences Pharmacochimie, Université Mohammed V-Agdal, BP 1014 Avenue Ibn Batout, Rabat, Morocco; d Department of Chemistry, University of Malaya, 50603 Kuala Lumpur, Malaysia

## Abstract

In the mol­ecule of the title compound, C_15_H_12_BrN_3_·H_2_O, the phenyl ring is coplanar with the imidazopyridine ring system [dihedral angle = 0.4 (1)°]. The water mol­ecule is disordered over two positions with occupancies of 0.58 (1) and 0.42 (1), and it is linked to the main mol­ecule *via* an O—H⋯N hydrogen bond.

## Related literature

For a related structure, see: Ouzidan *et al.* (2010 ▶).
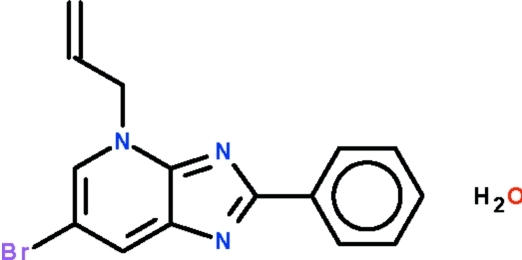


## Experimental

### 

#### Crystal data

C_15_H_12_BrN_3_·H_2_O
*M* *_r_* = 332.20
Triclinic, 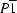

*a* = 7.4363 (1) Å
*b* = 9.4238 (1) Å
*c* = 11.0829 (2) Å
α = 68.076 (1)°
β = 74.637 (1)°
γ = 79.736 (1)°
*V* = 692.02 (2) Å^3^
*Z* = 2
Mo *K*α radiation
μ = 2.97 mm^−1^
*T* = 293 K
0.20 × 0.20 × 0.15 mm

#### Data collection

Bruker X8 APEXII area-detector diffractometer
Absorption correction: multi-scan (*SADABS*; Sheldrick, 1996 ▶) *T*_min_ = 0.588, *T*_max_ = 0.664
14314 measured reflections
3158 independent reflections
2791 reflections with *I* > 2σ(*I*)
*R*_int_ = 0.029

#### Refinement

*R*[*F* ^2^ > 2σ(*F* ^2^)] = 0.027
*wR*(*F* ^2^) = 0.078
*S* = 0.98
3158 reflections
203 parameters
6 restraints
H atoms treated by a mixture of independent and constrained refinement
Δρ_max_ = 0.25 e Å^−3^
Δρ_min_ = −0.39 e Å^−3^

### 

Data collection: *APEX2* (Bruker, 2008 ▶); cell refinement: *SAINT* (Bruker, 2008 ▶); data reduction: *SAINT*; program(s) used to solve structure: *SHELXS97* (Sheldrick, 2008 ▶); program(s) used to refine structure: *SHELXL97* (Sheldrick, 2008 ▶); molecular graphics: *X-SEED* (Barbour, 2001 ▶); software used to prepare material for publication: *publCIF* (Westrip, 2010 ▶).

## Supplementary Material

Crystal structure: contains datablocks global, I. DOI: 10.1107/S1600536810025122/ci5117sup1.cif

Structure factors: contains datablocks I. DOI: 10.1107/S1600536810025122/ci5117Isup2.hkl

Additional supplementary materials:  crystallographic information; 3D view; checkCIF report

## Figures and Tables

**Table 1:** Hydrogen-bond geometry (Å, °)

*D*—H⋯*A*	*D*—H	H⋯*A*	*D*⋯*A*	*D*—H⋯*A*
O1—H11⋯N3	0.83 (1)	2.14 (3)	2.887 (4)	149 (5)

**Fig. 1.**
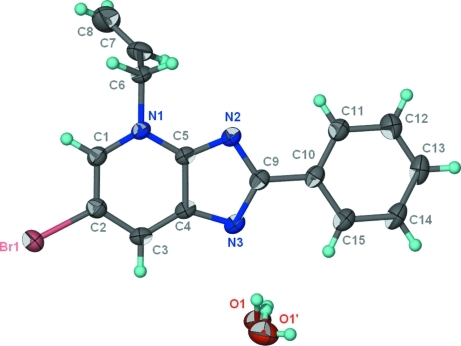
Thermal ellipsoid plot (Barbour, 2001) of the molecule of C15H12BrN3.H2O at the 50% probability level. H atoms are shown as spheres of arbitrary radii. The disorder in the water molecule is shown.

## supplementary crystallographic information

### Comment

The imidazo[4,5-*b*]pyridine unit is an important heterocyclic nucleus found in a large number of molecules in medicinal chemistry. Heterocycles derived from such compounds posess useful medicinal properties. Owing to their importance, strategies have been developed for their synthesis. The most popular synthetic approach involves the cyclocondensation of 2,3-pyridinediamine with carboxylic acid derivatives or on condensation with aldehydes. An earlier study reported the crystal structure of 4-benzyl-6-bromo-2-phenyl-4*H*-imidazo[4,5-*b*]pyridine (Ouzidan *et al.*, 2010), which was synthesized by using a much more convenient route. The synthesis is extended to the title compound (Scheme I and Fig. 1).

The imidazopyridine ring system is coplanar with the phenyl ring at the 2-position of the five-membered ring [dihedral angle = 0.4 (1) °].

### Experimental

To a solution 6-bromo-2-phenyl-1*H*-imidazo[4,5-*b*]pyridine (0.3 g, 1.09 mmol), was added a DMF (15 ml) solution of potassium carbonate (0.2 g, 1.42 mmol), tetra-*n*-butylammonium bromide (0.04 g, 0.1 mmol) and allyl bromide (0.11 ml, 1.31 mmol). Stirring was continued at room temperature for 12 h. The mixture was filtered and the solvent removed under reduced pressure. The residue was separated by chromatography on a column of silica gel with ethyl acetate-hexane (2:3) as eluent. Yellow crystals were isolated when the solvent was allowed to evaporate.

### Refinement

Carbon-bound H-atoms were placed in calculated positions (C–H = 0.93–0.97 Å) and were included in the refinement in the riding model approximation, with *U*(H) set to 1.2*U*_eq_(C). The water molecule is disordered over two positions in a 58 (1):42 (1) ratio. The H atoms were located in a difference Fourier map and were refined with distance restraints of O–H = 0.84 (1) Å and H···H 1.37 (1) Å; their U_iso_ values were tied to those of the oxygen atoms by a factor of 1.5.

### Crystal data

**Table:**

C_15_H_12_BrN_3_·H_2_O	*Z* = 2
*M**_r_* = 332.20	*F*(000) = 336
Triclinic, *P*1	*D*_x_ = 1.594 Mg m^−^^3^
Hall symbol: -P 1	Mo *K*α radiation, λ = 0.71073 Å
*a* = 7.4363 (1) Å	Cell parameters from 7204 reflections
*b* = 9.4238 (1) Å	θ = 2.3–27.2°
*c* = 11.0829 (2) Å	µ = 2.97 mm^−^^1^
α = 68.076 (1)°	*T* = 293 K
β = 74.637 (1)°	Prism, yellow
γ = 79.736 (1)°	0.20 × 0.20 × 0.15 mm
*V* = 692.02 (2) Å^3^	

### Data collection

**Table:**

Bruker X8 APEXII area-detector diffractometer	3158 independent reflections
Radiation source: fine-focus sealed tube	2791 reflections with *I* > 2σ(*I*)
graphite	*R*_int_ = 0.029
φ and ω scans	θ_max_ = 27.5°, θ_min_ = 2.5°
Absorption correction: multi-scan (*SADABS*; Sheldrick, 1996)	*h* = −9→9
*T*_min_ = 0.588, *T*_max_ = 0.664	*k* = −12→12
14314 measured reflections	*l* = −13→14

### Refinement

**Table:**

Refinement on *F*^2^	Primary atom site location: structure-invariant direct methods
Least-squares matrix: full	Secondary atom site location: difference Fourier map
*R*[*F*^2^ > 2σ(*F*^2^)] = 0.027	Hydrogen site location: inferred from neighbouring sites
*wR*(*F*^2^) = 0.078	H atoms treated by a mixture of independent and constrained refinement
*S* = 0.98	*w* = 1/[σ^2^(*F*_o_^2^) + (0.0529*P*)^2^ + 0.1091*P*] where *P* = (*F*_o_^2^ + 2*F*_c_^2^)/3
3158 reflections	(Δ/σ)_max_ = 0.001
203 parameters	Δρ_max_ = 0.25 e Å^−^^3^
6 restraints	Δρ_min_ = −0.39 e Å^−^^3^

### Fractional atomic coordinates and isotropic or equivalent isotropic displacement parameters (Å^2^)

**Table:**

	*x*	*y*	*z*	*U*_iso_*/*U*_eq_	Occ. (<1)
Br1	0.78351 (3)	0.73753 (2)	0.425170 (19)	0.05007 (9)	
O1	1.1389 (9)	0.1942 (6)	0.8855 (4)	0.0635 (16)	0.582 (14)
H11	1.028 (3)	0.229 (7)	0.903 (7)	0.095*	0.582 (14)
H12	1.208 (6)	0.237 (6)	0.908 (6)	0.095*	0.582 (14)
O1'	1.0501 (14)	0.1260 (12)	0.8987 (6)	0.081 (3)	0.418 (14)
H13	1.089 (17)	0.191 (10)	0.917 (11)	0.122*	0.418 (14)
H14	1.001 (16)	0.059 (9)	0.969 (6)	0.122*	0.418 (14)
N1	0.61186 (19)	0.78390 (15)	0.79149 (15)	0.0323 (3)	
N2	0.66816 (19)	0.61173 (15)	1.00435 (15)	0.0347 (3)	
N3	0.82874 (19)	0.41411 (16)	0.93153 (16)	0.0358 (3)	
C1	0.6432 (2)	0.80592 (19)	0.66074 (18)	0.0350 (3)	
H1	0.5976	0.8977	0.6035	0.042*	
C2	0.7415 (2)	0.6947 (2)	0.61055 (19)	0.0368 (4)	
C3	0.8123 (2)	0.55420 (19)	0.69201 (19)	0.0371 (4)	
H3	0.8780	0.4791	0.6576	0.044*	
C4	0.7798 (2)	0.53257 (18)	0.82533 (18)	0.0331 (3)	
C5	0.6797 (2)	0.65118 (18)	0.87503 (17)	0.0313 (3)	
C6	0.5091 (2)	0.90590 (19)	0.84527 (19)	0.0381 (4)	
H6A	0.4210	0.9672	0.7908	0.046*	
H6B	0.4386	0.8587	0.9352	0.046*	
C7	0.6408 (3)	1.0068 (2)	0.8464 (2)	0.0481 (5)	
H7	0.7367	0.9607	0.8917	0.058*	
C8	0.6310 (5)	1.1541 (3)	0.7888 (3)	0.0711 (7)	
H8A	0.5368	1.2037	0.7427	0.085*	
H8B	0.7180	1.2105	0.7933	0.085*	
C9	0.7604 (2)	0.46733 (18)	1.03315 (18)	0.0339 (3)	
C10	0.7799 (2)	0.37743 (19)	1.17031 (18)	0.0354 (4)	
C11	0.7034 (3)	0.4364 (2)	1.2724 (2)	0.0422 (4)	
H11A	0.6407	0.5342	1.2535	0.051*	
C12	0.7194 (3)	0.3511 (3)	1.4024 (2)	0.0498 (5)	
H12A	0.6678	0.3919	1.4699	0.060*	
C13	0.8118 (3)	0.2058 (3)	1.4313 (2)	0.0522 (5)	
H13A	0.8219	0.1481	1.5184	0.063*	
C14	0.8890 (3)	0.1467 (2)	1.3309 (2)	0.0509 (5)	
H14A	0.9520	0.0490	1.3505	0.061*	
C15	0.8741 (3)	0.2306 (2)	1.2012 (2)	0.0436 (4)	
H15	0.9269	0.1892	1.1342	0.052*	

### Atomic displacement parameters (Å^2^)

**Table:**

	*U*^11^	*U*^22^	*U*^33^	*U*^12^	*U*^13^	*U*^23^
Br1	0.06412 (15)	0.05036 (14)	0.03761 (13)	0.00253 (9)	−0.01310 (9)	−0.01926 (9)
O1	0.076 (3)	0.056 (2)	0.068 (2)	0.020 (2)	−0.0289 (19)	−0.0341 (17)
O1'	0.091 (5)	0.084 (5)	0.074 (3)	0.022 (4)	−0.021 (3)	−0.044 (3)
N1	0.0350 (7)	0.0260 (6)	0.0359 (8)	0.0023 (5)	−0.0108 (6)	−0.0108 (5)
N2	0.0360 (7)	0.0294 (7)	0.0370 (8)	0.0013 (5)	−0.0094 (6)	−0.0104 (6)
N3	0.0352 (7)	0.0288 (7)	0.0422 (8)	0.0013 (5)	−0.0112 (6)	−0.0107 (6)
C1	0.0373 (8)	0.0307 (8)	0.0371 (9)	0.0006 (6)	−0.0122 (7)	−0.0106 (7)
C2	0.0394 (8)	0.0368 (9)	0.0373 (9)	−0.0032 (7)	−0.0104 (7)	−0.0148 (7)
C3	0.0382 (8)	0.0321 (8)	0.0435 (10)	0.0008 (6)	−0.0084 (7)	−0.0181 (7)
C4	0.0307 (7)	0.0276 (7)	0.0418 (9)	0.0004 (6)	−0.0095 (7)	−0.0129 (7)
C5	0.0302 (7)	0.0274 (7)	0.0368 (9)	−0.0010 (6)	−0.0083 (6)	−0.0116 (6)
C6	0.0422 (9)	0.0305 (8)	0.0394 (9)	0.0086 (7)	−0.0097 (7)	−0.0143 (7)
C7	0.0513 (11)	0.0487 (11)	0.0530 (12)	0.0017 (8)	−0.0112 (9)	−0.0303 (9)
C8	0.101 (2)	0.0508 (13)	0.0645 (16)	−0.0212 (13)	−0.0088 (14)	−0.0227 (12)
C9	0.0293 (7)	0.0299 (8)	0.0402 (9)	−0.0027 (6)	−0.0087 (6)	−0.0085 (7)
C10	0.0318 (8)	0.0307 (8)	0.0407 (9)	−0.0048 (6)	−0.0107 (7)	−0.0058 (7)
C11	0.0443 (9)	0.0376 (9)	0.0419 (10)	−0.0011 (7)	−0.0118 (8)	−0.0101 (7)
C12	0.0521 (11)	0.0543 (12)	0.0419 (11)	−0.0061 (9)	−0.0123 (9)	−0.0132 (9)
C13	0.0524 (11)	0.0521 (12)	0.0427 (11)	−0.0107 (9)	−0.0178 (9)	0.0021 (9)
C14	0.0495 (11)	0.0370 (10)	0.0561 (13)	−0.0005 (8)	−0.0199 (9)	−0.0006 (9)
C15	0.0432 (9)	0.0348 (9)	0.0482 (11)	0.0000 (7)	−0.0120 (8)	−0.0093 (8)

### Geometric parameters (Å, °)

**Table:**

Br1—C2	1.8887 (19)	C6—C7	1.487 (3)
O1—H11	0.834 (10)	C6—H6A	0.97
O1—H12	0.838 (10)	C6—H6B	0.97
O1—H13	0.43 (12)	C7—C8	1.291 (3)
O1'—H11	0.97 (5)	C7—H7	0.93
O1'—H13	0.836 (10)	C8—H8A	0.93
O1'—H14	0.838 (10)	C8—H8B	0.93
N1—C1	1.346 (2)	C9—C10	1.470 (3)
N1—C5	1.354 (2)	C10—C11	1.389 (3)
N1—C6	1.489 (2)	C10—C15	1.396 (2)
N2—C5	1.322 (2)	C11—C12	1.388 (3)
N2—C9	1.372 (2)	C11—H11A	0.93
N3—C9	1.344 (2)	C12—C13	1.379 (3)
N3—C4	1.365 (2)	C12—H12A	0.93
C1—C2	1.375 (2)	C13—C14	1.374 (3)
C1—H1	0.93	C13—H13A	0.93
C2—C3	1.398 (2)	C14—C15	1.381 (3)
C3—C4	1.374 (3)	C14—H14A	0.93
C3—H3	0.93	C15—H15	0.93
C4—C5	1.433 (2)		
			
H11—O1—H12	110.6 (18)	H6A—C6—H6B	108.0
H12—O1—H13	101 (6)	C8—C7—C6	124.3 (2)
H11—O1'—H14	114 (9)	C8—C7—H7	117.8
H13—O1'—H14	110.2 (19)	C6—C7—H7	117.8
C1—N1—C5	119.78 (14)	C7—C8—H8A	120.0
C1—N1—C6	120.89 (14)	C7—C8—H8B	120.0
C5—N1—C6	119.29 (14)	H8A—C8—H8B	120.0
C5—N2—C9	101.46 (14)	N3—C9—N2	117.02 (16)
C9—N3—C4	103.09 (13)	N3—C9—C10	122.81 (15)
N1—C1—C2	120.95 (16)	N2—C9—C10	120.17 (16)
N1—C1—H1	119.5	C11—C10—C15	118.49 (18)
C2—C1—H1	119.5	C11—C10—C9	120.61 (16)
C1—C2—C3	122.00 (17)	C15—C10—C9	120.90 (17)
C1—C2—Br1	117.99 (14)	C12—C11—C10	120.79 (18)
C3—C2—Br1	120.01 (13)	C12—C11—H11A	119.6
C4—C3—C2	116.73 (15)	C10—C11—H11A	119.6
C4—C3—H3	121.6	C13—C12—C11	120.0 (2)
C2—C3—H3	121.6	C13—C12—H12A	120.0
N3—C4—C3	132.84 (15)	C11—C12—H12A	120.0
N3—C4—C5	106.85 (15)	C14—C13—C12	119.7 (2)
C3—C4—C5	120.29 (15)	C14—C13—H13A	120.1
N2—C5—N1	128.19 (15)	C12—C13—H13A	120.1
N2—C5—C4	111.57 (14)	C13—C14—C15	120.79 (19)
N1—C5—C4	120.24 (16)	C13—C14—H14A	119.6
C7—C6—N1	110.95 (14)	C15—C14—H14A	119.6
C7—C6—H6A	109.4	C14—C15—C10	120.2 (2)
N1—C6—H6A	109.4	C14—C15—H15	119.9
C7—C6—H6B	109.4	C10—C15—H15	119.9
N1—C6—H6B	109.4		
			
C5—N1—C1—C2	0.8 (2)	C1—N1—C6—C7	−91.40 (19)
C6—N1—C1—C2	178.59 (16)	C5—N1—C6—C7	86.45 (19)
N1—C1—C2—C3	0.4 (3)	N1—C6—C7—C8	124.1 (2)
N1—C1—C2—Br1	−178.60 (12)	C4—N3—C9—N2	0.46 (19)
C1—C2—C3—C4	−0.4 (3)	C4—N3—C9—C10	179.96 (15)
Br1—C2—C3—C4	178.57 (12)	C5—N2—C9—N3	0.11 (19)
C9—N3—C4—C3	177.66 (18)	C5—N2—C9—C10	−179.41 (14)
C9—N3—C4—C5	−0.79 (17)	N3—C9—C10—C11	−178.67 (16)
C2—C3—C4—N3	−178.94 (17)	N2—C9—C10—C11	0.8 (2)
C2—C3—C4—C5	−0.7 (2)	N3—C9—C10—C15	0.8 (2)
C9—N2—C5—N1	179.89 (16)	N2—C9—C10—C15	−179.71 (15)
C9—N2—C5—C4	−0.63 (17)	C15—C10—C11—C12	−0.3 (3)
C1—N1—C5—N2	177.63 (16)	C9—C10—C11—C12	179.18 (17)
C6—N1—C5—N2	−0.2 (3)	C10—C11—C12—C13	−0.1 (3)
C1—N1—C5—C4	−1.8 (2)	C11—C12—C13—C14	0.5 (3)
C6—N1—C5—C4	−179.69 (15)	C12—C13—C14—C15	−0.4 (3)
N3—C4—C5—N2	0.95 (19)	C13—C14—C15—C10	0.0 (3)
C3—C4—C5—N2	−177.74 (14)	C11—C10—C15—C14	0.4 (3)
N3—C4—C5—N1	−179.52 (14)	C9—C10—C15—C14	−179.12 (16)
C3—C4—C5—N1	1.8 (2)		

### Hydrogen-bond geometry (Å, °)

**Table:**

*D*—H···*A*	*D*—H	H···*A*	*D*···*A*	*D*—H···*A*
O1—H11···N3	0.83 (1)	2.14 (3)	2.887 (4)	149 (5)
O1—H12···N2^i^	0.84 (1)	2.41 (2)	3.229 (7)	165 (5)

Symmetry codes: (i) −*x*+2, −*y*+1, −*z*+2.
